# Hip Joint Torsional Loading Before and After Cam Femoroacetabular Impingement Surgery

**DOI:** 10.1177/0363546518815159

**Published:** 2018-12-31

**Authors:** K.C. Geoffrey Ng, Hadi El Daou, Marcus J.K. Bankes, Ferdinando Rodriguez y Baena, Jonathan R.T. Jeffers

**Affiliations:** * Department of Mechanical Engineering, Imperial College London, London, UK; † Department of Orthopaedics, Guy’s and St. Thomas’ NHS Foundation Trust, London, UK; ‡ Fortius Clinic, London, UK; Investigation performed at the Department of Mechanical Engineering, Imperial College London, London, UK

**Keywords:** cam, femoroacetabular impingement, capsule, robot, in vitro

## Abstract

**Background:**

Surgical management of cam femoroacetabular impingement (FAI) aims to preserve the native hip and restore joint function, although it is unclear how the capsulotomy, cam deformity, and capsular repair influence joint mechanics to balance functional mobility.

**Purpose:**

To examine the contributions of the capsule and cam deformity to hip joint mechanics. Using in vitro, cadaveric methods, we examined the individual effects of the surgical capsulotomy, cam resection, and capsular repair on passive range of motion and resistance of applied torque.

**Study Design:**

Descriptive laboratory study.

**Methods:**

Twelve cadaveric hips with cam deformities were skeletonized to the capsule and mounted onto a robotic testing platform. The robot positioned each intact hip in multiple testing positions: (1) extension, (2) neutral 0°, (3) flexion 30°, (4) flexion 90°, (5) flexion-adduction and internal rotation (FADIR), and (6) flexion-abduction and external rotation. Then the robot performed applicable internal and external rotations, recording the neutral path of motion until a 5-N·m of torque was reached in each rotational direction. Each hip then underwent a series of surgical stages (T-capsulotomy, cam resection, capsular repair) and was retested to reach 5 N·m of internal and external torque again after each stage. During the capsulotomy and cam resection stages, the initial intact hip’s recorded path of motion was replayed to measure changes in resisted torque.

**Results:**

Regarding changes in motion, external rotation increased substantially after capsulotomies, but internal rotation only further increased at flexion 90° (change +32%, *P* = .001, *d* = 0.58) and FADIR (change +33%, *P* < .001, *d* = 0.51) after cam resections. Capsular repair provided marginal restraint for internal rotation but restrained the external rotation compared with the capsulotomy stage. Regarding changes in torque, both internal and external torque resistance decreased after capsulotomy. Compared with the capsulotomy stage, cam resection further reduced internal torque resistance during flexion 90° (change −45%, *P* < .001, *d* = 0.98) and FADIR (change −37%, *P* = .003, *d* = 1.0), where the cam deformity accounted for 21% of the intact hip’s torsional resistance in flexion 90° and 27% in FADIR.

**Conclusion:**

Although the capsule played a predominant role in joint constraint, the cam deformity provided 21% to 27% of the intact hip’s resistance to torsional load in flexion and internal rotation. Resecting the cam deformity would remove this loading on the chondrolabral junction.

**Clinical Relevance:**

These findings are the first to quantify the contribution of the cam deformity to resisting hip joint torsional loads and thus quantify the reduced loading on the chondrolabral complex that can be achieved after cam resection.

Labral tears and groin pain resulting from cam-type femoroacetabular impingement (FAI) constitute a large portion of athletic hip injuries.^15,36,58,61^ The cam deformity is characterized by an enlarged, aspherical femoral head-neck and is associated with progressive hip pain and early joint degeneration,^2,33,43^ whereas mechanical impingement occurs when the femoral cam deformity obstructs the chondrolabral junction in flexion and rotation, resulting in limited mobility and adverse joint loading.^14,24,55^ Although previous pathoanatomic studies linked limited hip mobility primarily with the cam deformity, a growing population of athletes have asymptomatic cam morphologic features associated with preadolescent physical activity.^35,38,40,75,84,85^

Interest is emerging in how symptomatic FAI can be attributed to other causative anatomic and functional factors, in addition to the bony cam deformity.^6,10,21,52,54,68^ One such factor is the contribution of the capsular ligaments and their role in functional joint stability. Conventional hip preservation techniques involve either an arthroscopic or open surgical approach, accessing the anterosuperior head-neck deformity through an incised capsular portal. Although surgical cam resection aims to restore joint function^13,23,44,69,70^ and preserve the native hip,^3,9,32^ it is still unknown how the removal of the cam deformity influences hip joint loading. Moreover, it is unclear how capsular release and repair influence joint mechanics to balance functional mobility. Because joint-preserving surgery for FAI is a recurring subject of interest for athletic hip injuries, these topics warrant closer examination.

A subject of debate regards whether an approach that entails capsulotomy alone can restore hip mobility to levels similar to those achieved by resection of the cam deformity or whether capsular repair is necessary after cam resection.^¶^ A few recent biomechanical studies examined the effects of a capsulotomy and subsequent capsular repair on cadaveric hips.^1,62,83^ However, none of the studies examined changes in torque restraint of pathological hips, which would help characterize each surgical procedure’s effect on joint loading and stability. Previous computational models also estimated the adverse loading mechanics attributed to cam FAI, although none of the models included the contributions of the capsular ligaments. More recently, increasing interest has turned to implementation of robotic testing platforms to examine soft tissue contributions and various implants on knee^4,27,41,81^ and hip joint mechanics^28,34,62,72^; however, the effects of surgical stages on hips with cam morphologic features have yet to be examined. Therefore, the purpose of our study was to quantify the contributions of the capsule and cam deformity to hip joint mechanics and investigate the influence of capsular repair during surgical intervention of FAI.

## Methods

This descriptive laboratory study involved in vitro cadaveric methods that investigated the effects of surgical stages on hip joint morphologic features and capsular mechanics. The investigations were conducted ethically in conformity with research principles, and the study protocol was approved by the institution’s research ethics boards (No. R14088-1A).

### Specimen Preparation

**Figure 1.**
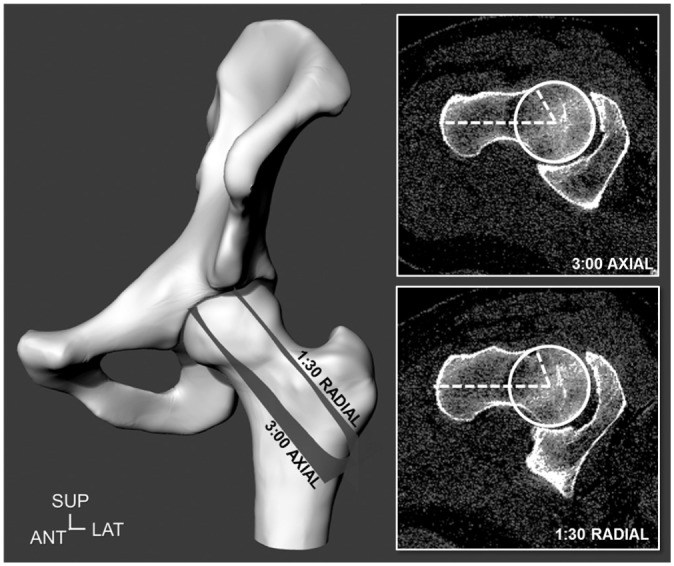
Hip joints were included if they indicated a cam deformity on computed tomography (CT) data, as defined by an alpha angle greater than 50.5° in the axial 3:00 plane or greater than 60° in the radial 1:30 plane on the depicted left hip joint model and CT imaging plane. ANT, anterior; LAT, lateral; SUP, superior.

Twelve fresh-frozen cadaveric hips were acquired from a tissue bank and included in this study. The hips were acquired as pairs from 6 male specimens with bilateral cam deformities (mean age ± SD, 45 ± 9 years; mean body mass index [BMI] ± SD, 24 ± 3 kg/m^2^). Each hip was treated as an independent case, as there was no history that indicated which side was more symptomatic or which was the dominant leg. Specimens were screened for age, BMI, and sex (age <60 years, BMI <30 kg/m^2^, male), as the cam deformity has been statistically more prevalent in younger, athletic males.^2,67^ Before the joints of interest were denuded and truncated, each intact cadaveric body was positioned on an imaging table, in a standard supine position with natural lordosis, corrected pelvic obliquity, and toes together pointing anteriorly. A conventional computed tomography (CT) scanner (Somatom Perspective; Siemens) was used to image the intact pelvic region (iliac crest to lesser trochanter) and knees, with 512 × 512 resolution, 0.6-mm slice thickness, 130 kVp, and 0.772-mm pixel spacing. Each specimen’s CT data were evaluated for multiple anatomic femoral, acetabular, and spinopelvic parameters, according to established measurement protocols (Table 1).^52-54^ Specimens were included if they indicated a cam deformity on CT data (ie, axial 3:00 alpha angle >50.5° or radial 1:30 alpha angle >60°, in their respective clock-face positions; Figure 1)^35,64^ and were excluded if they hadany other hip abnormalities (eg, slipped capital femoral epiphysis, Legg-Calvé-Perthes, dysplasia, overcoverage), musculoskeletal disorders, cancer that metastasized to the spine or hip region, or history of lower limb or spinal arthritis, surgery, or trauma. All superficial skin, fat, and muscles were removed from the specimen, skeletonizing each set of pelvis and femurs to bone and leaving hip joints intact to the ligamentous capsule.

**Table 1:** Descriptive Demographic and Anatomic Parameters of the Specimens^*a*^

Parameter	Measurement
Specimens, n	12
Side, left/right, n	6/6
Age, y	45 ± 9
Body mass index, kg/m^2^	24 ± 3
Cam deformity parameter	
Axial 3:00 alpha angle, deg	63 ± 6
Radial 1:30 alpha angle, deg	74 ± 3
Femoral head-neck offset, mm	4 ± 2
Neck angle parameter	
Femoral neck-shaft angle, deg	128 ± 4
Medial proximal femoral angle, deg	78 ± 5
Torsion/version	
Femoral torsion, deg	10 ± 5
Acetabular version, deg	26 ± 6
Acetabular coverage	
Center edge angle, deg	34 ± 5
Spinopelvic parameter	
Pelvic incidence, deg	54 ± 13

a Values are expressed as mean ± SD except for numbers of specimens.

### Robotic Testing Platform

Each specimen’s anatomic pelvic landmarks were digitized to establish a reference frame^28^ by use of an optical tracking system (Polaris; Northern Digital Inc). Each specimen was then separated into 2 hemi-pelvises (sectioned at the sacroiliac and pubic symphysis joints) and ipsilateral hip joint, truncating the proximal third of the femur at the diaphysis. The proximal femur and hemi-pelvis were securely potted into custom cylindrical and box pots, respectively, with polymethyl methacrylate,^28^ and aligned to the International Society of Biomechanics (ISB) recommendations for joint coordinate systems.^78,82^

The pelvic pot was then securely fixed in an inverted position, onto a fixed testing platform, with the femoral component free in rotation. Rigid body marker arrays (X-Y arrays; Brainlab AG) were attached to the pelvis and femur and then digitized to determine the transformation matrices between the associated marker arrays, anatomic landmarks, and global coordinate system.^28^ To determine the initial hip joint center of rotation and also minimize tissue hysteresis, the optical tracking system captured the marker arrays as the femur was manually rotated multiple times, in a combined star-circumduction motion.^17^ A sphere fitting, least squares approach was used to define the hip joint center toward the femoral pot.^28^

**Figure 2.**
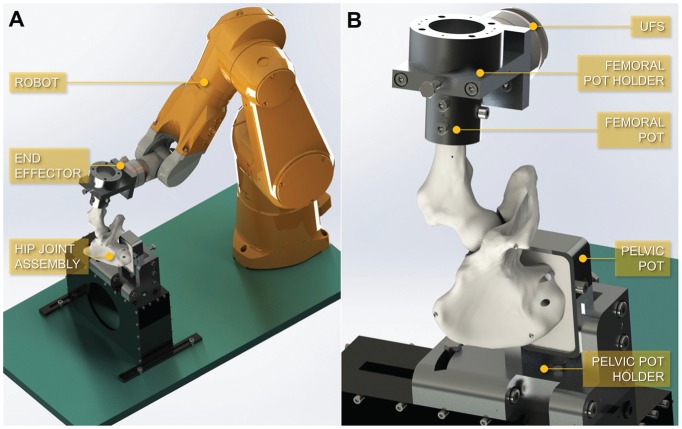
(A) The robotic testing platform, composed of a 6 degrees of freedom industrial robot, with the hip joint assembly attached to the platform fixture and robot end effector. (B) The hip joint assembly (left-sided hip with capsule not depicted), placed in an inverted position, with the femoral and pelvic pots secured in their respective holders, and with a universal force-moment sensor (UFS) between the end effector and the femoral component.

The femoral pot was then securely mounted onto the end effector of a 6 degrees of freedom industrial robot (TX90; Stäubli) equipped with a universal force-moment sensor (Omega85; ATI Industrial Automation) (Figure 2). Initially, the femoral axis was aligned orthogonally to the anterior to posterior superior iliac spine axis; however, this initial alignment position generated substantial residual forces in several of the hip joints, as measured by the universal sensor. Because anterior pelvic tilt is commonly required for neutral hip joint positioning and sagittal mobility,^12,46,54^ the robot permitted each hip’s femoral component to flex and move until forces and torques were neutralized, similar to previous knee mechanics and soft tissues studies,^41^ to establish the neutral standing position (anterior pelvic tilt, 2°-14°).

Since the femoral head is naturally conchoidal and the cam deformity is expected to exacerbate the asphericity, it would not be correct to assume that the center of rotation is fixed about a single point.^49^ Thus, a hybrid force-position controller decoupled the control of the 6 degrees of freedom in force or displacement, permitting a naturally unconstrained hip joint center.^28^ The robot’s coordinate system for control was located at the hip joint center, with its rotational axes defined by ISB recommendations.

### Testing Positions

**Figure 3.**
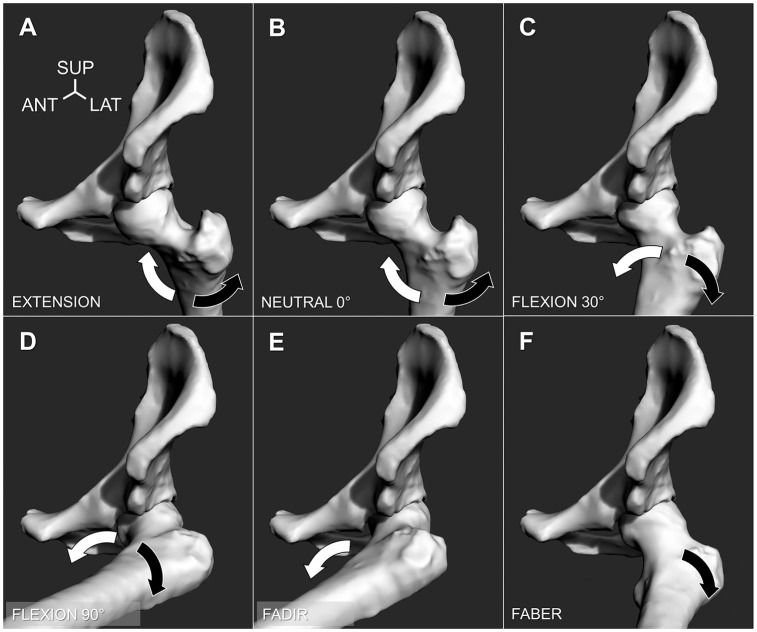
The hip positions considered in the study (depicted left-sided): (A) extension; (B) neutral 0°; (C) flexion 30°; (D) flexion 90°; (E) flexion-adduction, internal rotation (FADIR); and (F) flexion-abduction, external rotation (FABER). The robot performed maximum internal rotation (white arrow) and external rotation (black arrow), capturing the path of motion until a 5-N·m torque was reached. (For clarity, the hip capsule is not depicted.) ANT, anterior; LAT, lateral; SUP, superior.

The robot positioned each hip joint in 6 testing positions, which included 4 sagittal plane angles and 2 clinical impingement scenarios: (1) full extension, (2) neutral 0° (standing position), (3) flexion 30° (heel strike position), (4) flexion 90° (sitting position), (5) flexion-adduction and internal rotation (FADIR), and (6) flexion-abduction and external rotation (FABER). In each position, the robot performed applicable internal-external rotations of the intact hip, until a torque resistance of 5 N·m was measured in each rotational direction, for 2 cycles (Figure 3; see online Video Supplement). A compressive load of 5 N was applied to keep the hip joint in contact throughout testing. As the primary focus was the capsular mechanics and passive range of motion, a relatively small load was preferred so as not to compromise the joint and tissues or risk any damage to them. For the FADIR and FABER positions, the robot first applied 5 N·m of adduction and abduction, respectively; flexion 90°; and then 5 N·m of internal and external rotational torque, respectively. Hips were rotated slowly at a fixed angular speed (internal-external rotation, 0.8 deg/s; abduction-adduction, 1.6 deg/s) for all tests to ensure that any rate dependence did not influence results. Each path of internal-external rotational motion was recorded and stored.

### Testing Stages

**Figure 4.**
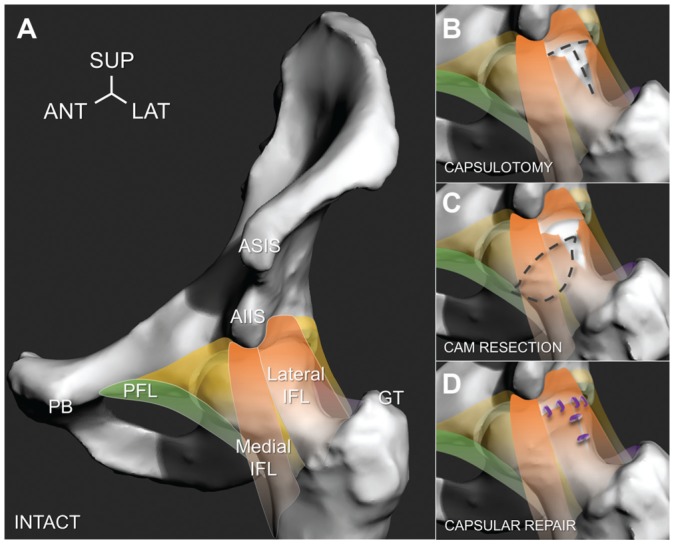
Four testing stages were evaluated, as depicted on a left-sided hip. (A) Intact hip with cam deformity, indicating lateral and medial branches of the iliofemoral ligament (IFL, orange), pubofemoral ligament (PFL, green), encapsulating tissue (yellow), anterior superior iliac spine (ASIS), anterior inferior iliac spine (AIIS), pubis (PB), and greater trochanter (GT) for reference. (B) Capsulotomy, where the lateral iliofemoral ligament was incised to create a T-capsulotomy (dashed lines). (C) Cam resection, where the anterior femoral head was accessed through the incised capsule and the cam deformity was resected (dashed lines). (D) Capsular repair, where the incised interportal and vertical limb portal were closed by use of interrupted sutures (purple knots). ANT, anterior; LAT, lateral; SUP, superior.

After the intact hip joint was tested as the first stage (Figure 4A), each hip underwent a series of surgical procedures. As the pelvic pot holders were fixed and the robot end effector was able to return to its initial starting positions, the hip was consistently repositioned into the robot and retested after each surgical stage (error <0.1 mm).^27,28^ In the second stage, a T-capsulotomy incision was performed to the lateral iliofemoral capsular ligament of each hip, creating an interportal and vertical limb portal (Figure 4B). The interportal was incised 5 mm away from the capsulolabral complex and was within the lateral iliofemoral ligament’s width (Table 2). The vertical limb portal further exposed the anterosuperior cam deformity but was carefully performed so as not to disrupt the zona orbicularis. In the third stage, a 3-dimensional preoperative plan was established using each hip’s CT data to assess the size and location of the cam deformity and to predict the amount of resection needed. The cam deformity was resected by use of a rotary burring tool (Dremel 4000; Bosch) through the T-capsulotomy portal (Figure 4C). Caution was taken not to overresect the femoral head (ie, deep “cookie bite” or proximal concavity), and cam removal was deemed satisfactory with confirmed clearance during internal rotation. In the fourth stage, the lateral iliofemoral ligament was repaired through use of simple, interrupted sutures (No. 2-0 Vicryl; Ethicon Inc) to close the interportal (4 sutures) and vertical limb incisions (2 sutures), without capsular plication (Figure 4D). All surgery was performed by the senior orthopaedic surgeon (M.J.K.B.), and the specimens were frequently sprayed with water during testing to maintain the viability of the tissues.

**Table 2:** Width, Length, and Thickness of the Lateral Iliofemoral Ligament and Capsulotomy Incisions, at Neutral Position^*a*^

Parameter	Measurement
Preoperative intact width	33 ± 2
Interportal incision width	27 ± 3
Preoperative intact length	55 ± 3
Vertical limb incision length	23 ± 5
Postoperative thickness	8 ± 2

a Data reported as mean ± SD, mm.

### Changes in Range of Motion and Torque Resistance

The robot retested the hip after each surgical procedure in 2 ways. (1) To examine changes in internal-external rotations after each surgical stage, the robot applied a torsional load of 5 N·m and measured any changes in rotational position. Thus, the robot permitted the hip to find a new passive path of motion with an unconstrained hip joint center. (2) To examine changes in torque resistance, the robot replayed the intact hip’s recorded path of motion (ie, position control for playback) and measured the difference in torque resisted by the hip. This was performed after the capsulotomy and cam resection stages. The intact hip’s motions were not replayed for the capsular repair stage, as the repair altered the capsular tension and prevented the hip from following its initial intact paths. During (1) load and (2) position control testing, the peak amplitude of rotation (degrees) and peak torque resistance (N·m) were recorded and averaged for each position. Upon completion of testing, each specimen underwent a second CT scan to evaluate the completeness of the cam resection (Table 3).

**Table 3:** Comparison of Cam Deformity Parameters Before and After Resection^*a*^

Parameter	Preoperative Condition	Postoperative Condition
Axial 3:00 alpha angle, deg	63 ± 6	45 ± 4^*b*^
Radial 1:30 alpha angle, deg	73 ± 3	55 ± 3^*b*^
Femoral head-neck offset, mm	4 ± 2	7 ± 2^*c*^

a Values are expressed as mean ± SD.

b Significant difference compared with preoperative condition (*P* < .01).

c Significant difference compared with preoperative condition (*P* < .05).

Statistical analyses were performed with statistics software (SPSS version 24; IBM). One-way, repeated-measures analysis of variance was used to examine the effects of surgical stage on within-subject differences in internal-external torque resistance and internal-external amplitude of rotation, with Bonferroni corrections (95% CI). Paired-sample *t* tests were used to compare any detected differences between surgical stages, with Cohen *d* to indicate small (*d* > 0.2), medium (*d* > 0.5), and large effects (*d* > 0.8). A sample size calculator (G*Power 3.1.9.3; Heinrich-Heine-Universität Düsseldorf, Germany) determined that the acceptable sample size was 12 in order to seek 80% of statistical power and detect a large effect size.

## Results

### Changes in Range of Motion

All intact hip joints were tested at each position, reaching 5 N·m of internal and external torque resistance. During load control testing (ie, reapplying 5 N·m to measure changes in rotational motion), external rotation increased substantially after the capsulotomy, compared with the intact hip, and indicated medium to large effects, more notably at flexion 30° (increase, +10°; change, 30%; *P* < .001, *d* = 0.78), flexion 90° (increase, +8°; change, 21%; *P* = .015, *d* = 0.75), and FABER (increase, +9°; change, 41%; *P* < .001, *d* = 0.94). The capsulotomy had small to medium effects on internal rotation at extension (increase, +7°; change, +31%; *P* < .001, *d* = 0.55) and neutral 0° (increase, +5°; change; +19%, *P* < .001, *d* = 0.39) as well as on external rotation at extension (increase, +4°; change, +24%; *P* = .016, *d* = 0.43).

**Figure 5.**
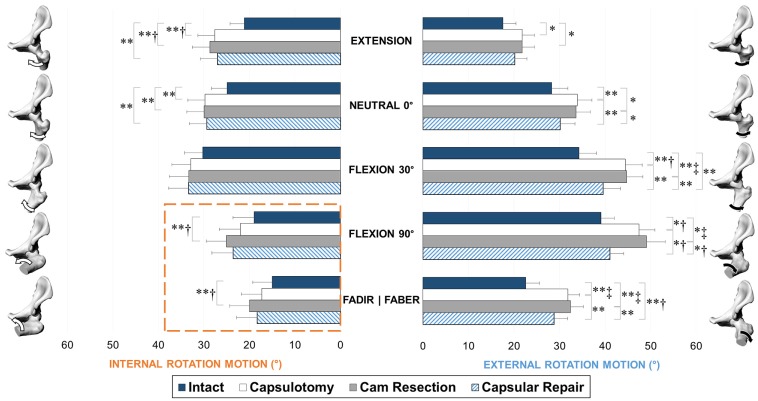
Range of motion in internal (left) and external (right) rotation, at each stage of testing: intact, capsulotomy, and cam resection, reported as mean and standard error. Significant differences: *P < .05 and **P < .01. Effect sizes: ^†^*d* > 0.5 and ^‡^*d* > 0.8. The hip models and arrows represent the testing positions during internal and external rotations. Cam resection further increased internal rotation in the flexion 90° and FADIR positions (highlighted within dashed lines). FABER, flexion-abduction and external rotation; FADIR, flexion-adduction and internal rotation.

After the cam resection stage, internal rotation increased substantially only at the flexion 90° (increase, +6°; change, +32%; *P* = .001, *d* = 0.58) and FADIR positions (increase, +5°; change, +33%; *P* < .001, *d* = 0.51) compared with the intact stage (Figure 5). No other differences were noted between the capsulotomy and cam resection stages in external or internal rotation. Subsequent capsular repair provided marginal restraint in internal rotation but helped restore external rotations at the neutral 0°, flexion 30°, flexion 90°, and FABER positions toward, but never reaching, the values of the intact hip (Figure 5).

### Changes in Torque Resistance

**Figure 6.**
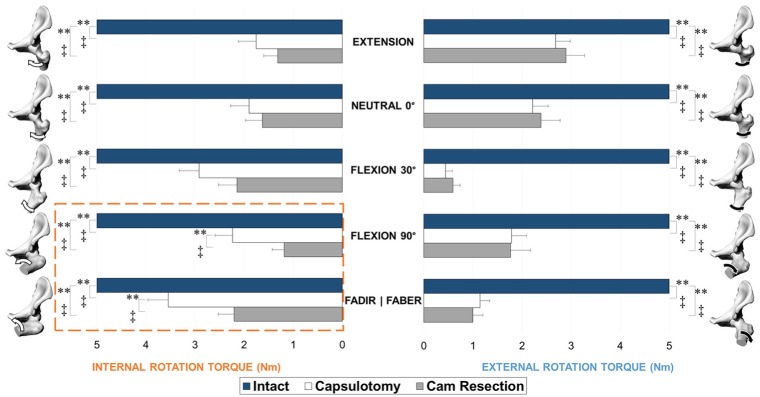
Torque restraint in internal (left) and external (right) rotation, at each stage of testing: intact, capsulotomy, and cam resection, reported as mean and SE. Significant differences: **P < .01. Effect sizes: ^‡^*d* > 0.8. The hip models and arrows represent the testing positions during internal and external rotations. Cam resection further decreased internal torque restraint in the flexion 90° and FADIR positions (highlighted within dashed lines). FABER, flexion abduction and external rotation; FADIR, flexion-adduction and internal rotation.

During position control testing (ie, playback of the intact hip’s path of motion to measure changes in torque restraint), both internal and external torsional resistance decreased for all testing positions after the capsulotomy stage, compared with the intact hip (*P* < .01, *d* > 0.8). However, after the cam resection stage, internal torsional resistance further decreased in only flexion 90° (decrease, –1.0 N·m; change, –45%; *P* < .001, *d* = 0.98) and FADIR (decrease, –1.3 N·m; change, –37%; *P* = .003, *d* = 1.0), compared with the capsulotomy stage (Figure 6). The cam deformity accounted for 21% in flexion 90° and 27% in FADIR relative to the torsional resistance of the intact hip. No differences were found in external torsional resistance after cam resections compared with the capsulotomy stage.

## Discussion

To avoid suboptimal clinical outcomes and potential iatrogenic instability, it is imperative to understand the effects of hip preservation surgery. Surgical injury to the soft tissues must be minimized to ensure that athletes can return to competition quickly and safely. This was the first study to use cadaveric hips with cam morphologic features to investigate the effects of the stages of hip preservation surgery for cam FAI (ie, capsulotomy, cam resection, capsular repair) on functional range of motion and resistance to applied torsional load. The most important finding in this study was that although the capsulotomy procedure was responsible for substantial overall changes in joint restraint, the cam deformity was responsible for 21% to 27% of the intact torsional resistance in flexion and internal rotation. Therefore, surgical cam resection would remove this load at the chondrolabral junction.

During load control testing (ie, changes in range of motion), external rotation increased substantially for all hip positions after the capsulotomy stage, which was comparable with findings of previous biomechanical studies that examined healthy control hips.^1,62,83^ During hip extension, cam resections did not affect range of motion or torque restraint, which suggests that any limited mobility at hip extension may be due to other pathoanatomic characteristics.^6,54^ However, resecting the cam deformity increased internal rotation compared with the intact case at the flexion 90° and FADIR positions, supporting the theory that removing the deformity would improve range of motion.^13,44,69,70^ It was important to examine changes in motion and torsional resistance after the capsulotomy stage and then again after the cam resection stage, as this isolated the contributions of the cam deformity and provided the amount of residual capsular restraint. We conducted the study in a stepwise manner, collecting the data of the intact hip before the capsulotomy, after the capsulotomy procedure (before the cam deformity was removed), and then after the cam deformity was removed. By doing so, we were able to observe the effects of the capsulotomy-only approach and, subsequently, were able to isolate the contributions of the cam morphologic feature toward internal and external rotational resistance. Interestingly, external rotational motion and torques were unaffected after the cam resections and did not change in FABER. This contradicts earlier suggestions that Drehmann sign or FABER tests can indicate cam FAI.^5,66,76^ Rather, this suggests that during physical examination, FABER can help indicate capsular tightness and soft tissue irritations (eg, due to psoas or iliocapsularis impairment) secondary to the cam deformity^30^ but not directly for cam FAI.

During position control testing (ie, changes in torque restraint), the advantage of the robotic testing platform was the ability to accurately play back the initial intact hip’s recorded path of motion after each subsequent surgical stage.^27,28,41^ This protocol was previously developed to examine soft tissue contributions to knee joint stability.^27,41^ However, the protocol was yet to be implemented for hip joint mechanics and, thus, now provides a benchmark for functional torque restraint attributed to cam FAI. Also, to our knowledge, previous in vitro cadaveric hip joint studies did not account for spinopelvic alignment or natural pelvic tilt. Legaye et al^46^ reported a pelvic tilt of 11.9°± 6.6° for men and 10.3°± 4.8° for women, whereas Boulay et al^12^ reported a pelvic tilt of 11.96°± 6.44°, values that were similar to our range of initial pelvic tilt values. By disregarding a more natural pelvic tilt and posture, several of the previous cadaveric hip joint studies may have biased their starting “neutral position” (hip flexion of 0°) and would have likely tested the hip in slight extension, resulting in a tighter hip and smaller range of motion. Our study indicated that the lateral iliofemoral ligament played a predominant role in joint stability, as both internal and external torsional resistance substantially decreased after capsulotomies. However, after the cam resection stage, internal torsional resistance further decreased at hip flexion positions of flexion 90° and FADIR. Our data indicated that 21% to 27% of the torsional resistance experienced by the intact hip in these hip positions was caused by the cam deformity pressing against the chondrolabral junction. Removing the cam, therefore, can mitigate adverse loading to the anterosuperior chondrolabral junction.^20,45,55,57^ This coincided with previous finite element models that simulated the effects of cam FAI on adverse loading, where hip models with large cam deformities demonstrated impingement risks and stresses at higher flexion angles.^20,37,57^ This finding also supports that removing the cam deformity can be beneficial to alleviate hip joint stresses,^56^ subchondral bone densities,^9^ and cartilage degeneration^3^ in comparison with healthy control hips.

While some studies opted to leave the capsule unrepaired and others advocated partial or full repairs,^1,16,26,31,47,60,74^ we compared both unrepaired (ie, cam resection stage) and repaired conditions. Recently, Wuerz et al^83^ examined an older cohort of cadaveric specimens (age, 67 ± 23 years) with higher acetabular overcoverage (center edge angle, 48.9°± 7.6°) and no cam deformity. The investigators examined only a neutral flexion position but reported similar angular displacements with their capsulotomy and repair stages. Similarly, Philippon et al^62^ used a robotic testing platform to examine capsulotomy and repair techniques on older cadaveric male specimens (mean age, 51 years; range, 38-65 years) with no known injuries or diseases and reported similar rotational restraints after full T-capsulotomy closures.^62^ In an imaging study, Strickland et al^74^ recently reported that both repaired and unrepaired interportal capsulotomies healed postoperatively at 24 weeks. Our capsular repair provided marginal restraint in internal rotation but restrained external rotations compared with the capsulotomy values. Although full capsular repairs could be effective for larger capsulotomies or overresected cams,^31,79^ closures should always be performed with extreme caution so as to not overtighten the capsule and exchange instability for joint tightness. The translations of the hip joint center of rotation^17,73^ should be examined in the future to help characterize microinstability over the series of surgical stages. Furthermore, the balance between capsular repair and plication should be further examined to establish objective measures to minimize iatrogenic instability.

Our study had certain limitations to note. First, our cohort consisted of young male specimens, which made it difficult to compare our study with previous cadaveric studies that examined older specimens. However, the cam deformity is more prevalent in younger males,^2,67^ and thus our cohort may be the most representative of cam FAI and capsular joint mechanics using in vitro cadaveric methods. We included 12 hips in our study, which was higher than many previous acceptable sample sizes that tested cadaveric hip specimens (n = 3-10 hips).^1,34,62,77,83^ However, including female specimens would further stratify the effect of anatomic structures on the pathological variances in joint mechanics, and a larger sample size would further increase the predictive power of the statistical models. Second, muscles were not included in the experiments, as we focused on the contributions of the bony deformity and capsular ligament to torque restraint and range of motion. As muscles provide a balance of passive and dynamic joint torque and stability,^51^ it would be important to further examine the effects of the surrounding muscles on microinstability.^39,50^ Third, it was not known whether the individuals who provided their hips for this study experienced any clinical symptoms of FAI that would have qualified them as candidates for hip preservation surgery. Interestingly, from the anatomic measurements, 1 hip had a small femoral neck-shaft angle (121°) and 4 hips had larger spinopelvic incidence angles (61°-76°), which were anatomic characteristics associated with symptomatic cam FAI (secondary to the cam deformity).^7,10,52,54,65^ All lateral iliofemoral ligaments were also relatively thick, as measured after the capsulotomy. This finding coincided with previous imaging studies that found thicker anterosuperior capsules in symptomatic cam FAI^63,80^ and suggested that our cohort was representative of individuals at risk of symptoms. Fourth, we performed the T-capsulotomy instead of an interportal-only capsulotomy. As our specimens were male hips with large cam morphology, the T-capsulotomy provided greater visualization and access to the joint space.^1,18^ Our interportal and vertical limb incisions were conservative, in comparison with conventional practice, and were more similar to a half-T.^59^ Previous biomechanical studies showed marginal differences in rotational restraint between unrepaired interportal and T-capsulotomies,^1,62^ and we further postulated that a small T-capsulotomy would not disrupt the structural integrity of the capsule, as the small vertical limb aligns with the lateral iliofemoral ligament’s fiber direction.^71^ Fifth, a relatively small compressive load was applied to the hip joint so as to not risk any damage to the joint and tissues. The minimal load demonstrated the passive range of motion and resistance of the hip joint capsule and cam, similar to what would be observed during a physical examination (ie, supine patient performing FADIR and FABER on a bench). Sixth, the cadaveric study examined the contributions of the capsule and cam deformity at time zero (ie, during and immediately after surgery); as such, the study did not consider the effects of subsequent healing and stabilization on range of motion and torque restraint.

Surgical intervention should continue to focus on cam resection for individuals in whom nonoperative treatments have failed or who have secondary pathoanatomic characteristics (eg, coxa vara, femoral anteversion, acetabular crossover, high spinopelvic incidence angles).^6,7,10,42,52,56^ Emphasis should be placed on proper patient selection in efforts to improve patient satisfaction and functional outcomes, as not all athletes have complete symptom resolution from surgery. Individuals who do not experience limited or painful internal rotation (ie, FADIR) but restricted external rotation (ie, FABER) could elect for nonsurgical management and further hip mobilization. Given that adverse loading to the chondrolabral junction and acetabular subchondral bone leads to hip joint degeneration, it would be crucial to elucidate the amount of capsular release, cam resection, and capsular repair needed without compromising hip mobility and function. The capsule played a predominant role in joint constraint; however, the cam deformity was responsible for a substantial amount of torsional resistance during hip flexion and internal rotation by pressing on the chondrolabral junction. It has long been suggested that surgical cam resection can restore hip motion and alleviate pain. This study demonstrated that, in flexion and internal rotation, the cam transmits 21% to 27% of the total load to the chondrolabral junction. Removing the cam removes this adverse loading.

## Footnotes

¶ References 8, 11, 18, 19, 22, 25, 29, 31, 48, 60.
